# Comparison of accommodation and vergence parameters in early and late-onset myopic adults

**DOI:** 10.1186/s12886-025-04543-5

**Published:** 2025-12-04

**Authors:** Shravya, Salai Dhavamathi Janarthanan, Vijaya Pai H

**Affiliations:** 1 Department of Optometry, Manipal College of Health Professions, Manipal Academy of Higher Education, Manipal, Karnataka India; 2 Department of Ophthalmology, Kasturba Medical College, Manipal Academy of Higher Education, Manipal, Karnataka India

**Keywords:** Early-onset myopia, Late-onset myopia, Accommodation, Vergence

## Abstract

**Significance:**

Myopia is a growing public health concern, and understanding the visual function differences among adults with different ages of myopia onset is essential for effective management. This study demonstrated that late-onset myopic adults have significantly reduced accommodative facility and lower fusional vergence amplitudes at distance compared to early-onset myopes. These findings highlight that the age of myopia onset influences accommodative and binocular vision behavior in adulthood, emphasizing the need for individualized clinical management strategies such as accommodative and vergence training in late-onset myopia.

**Purpose:**

This study aimed to assess and compare the accommodation and vergence parameters in early and late-onset myopic adults.

**Methods:**

This cross-sectional observational study was carried out at the Department of Ophthalmology, Kasturba Medical College and Optometry Clinic, Manipal. A total of 60 participants aged 18-35 years with mild to moderate defined as a spherical equivalent refractive error of –0.50 D to -6.00D were included in the study. A comprehensive eye examination was done to confirm eligibility criteria. Those who met the inclusion criteria were categorised into two groups: one with those who had early-onset myopia (EOM) and another with late-onset myopia (LOM). Accommodation and vergence parameters such as the amplitude of accommodation, accommodative facility, accommodative response, AC/A ratio, near point of convergence, positive fusional vergence, negative fusional vergence and vergence facility were measured.

**Results:**

A total of 60 participants (30 EOM and 30 LOM) were enrolled in the study. The mean age at presentation of the early-onset myopic individual was 21.8± 2.3, and the late-onset myopic individual was 21.9± 2.6. There was a statistically significant difference between EOM and LOM in both monocular and binocular accommodative facility, negative fusional vergence and positive fusional vergence for distance. Accommodative facility was reduced in late-onset myopes compared with early-onset myopes (OD: 8.5 ± 4.7 vs. 12.5 ± 4.5 cpm, *p* < 0.001; OS: 8.5 ± 4.1 vs. 12.0 ± 5.0 cpm, *p* = 0.001; OU: 9.2± 4.8 vs. 12.8± 4.3 cpm, *p* = 0.002;). Late-onset myopes also demonstrated lower negative fusional vergence at distance (break: 7.0 ± 5.5 vs 10.0 ± 4.0 PD, *p *= 0.002; recovery: 6.0 ± 5.5 vs 8.0 ± 4.0 PD, *p *= 0.005;) and positive fusional vergence at distance (break: 9.0 ± 8.0 vs 12.0 ± 11.0 PD, *p *= 0.018; recovery: 7.0 ± 8.0 vs 10.0 ± 11.0 PD, *p *= 0.024). Other parameters showed no significant difference between EOM and LOM.

**Conclusions:**

This study concluded that late-onset myopic individuals have reduced accommodative facility and lower negative and positive fusional vergence for distance compared to early-onset myopic individuals. These findings indicate that the age of myopia onset may influence visual performance, and the consideration of its characteristics of onset should be included in clinical evaluation and treatment.

## Key message

**What is known:**

• Myopic individuals often exhibit altered accommodative and vergence functions compared to emmetropes.

• Accommodation and vergence dysfunctions have been widely studied in children, but limited data exist comparing adults with early and late-onset myopia.

**What is new:**

•This study demonstrates that late-onset myopes have significantly reduced monocular and binocular accommodative facility compared to early-onset myopes.

• It reveals that late-onset myopic adults also show lower negative and positive fusional vergence at distance.

• These findings suggest that the age at which myopia develops may influence how the accommodative and vergence systems function in adulthood. Understanding these differences can help clinicians tailor management strategies.

## Introduction

Myopia, one of the most common refractive errors, is defined as a spherical equivalent refractive error of ≤ − 0.50 diopters (D) [1]. It is a major public health concern due to its high prevalence across the world. It is estimated that 49.8% population of the world will become myopic by 2050 [1]. In India, the current prevalence among children aged 5–15 years is reported to be 7.5% [2].

Myopia can be classified based on various factors, including etiology, degree of myopia, clinical entity, and age of onset. Based on the age of onset, it can be classified as early-onset myopia (EOM) and late-onset myopia (LOM). EOM is having the first spectacle or contact lens prescription before 15 years of age, and LOM is having the first refractive correction at 15 years of age or older [3].

EOM, often referred to as school-age myopia, is affected by both gene expression and environmental factors [4]. In contrast, LOM or adult-onset is usually related to prolonged near work and often restricted to low or moderate levels [5]. These differences suggest that the degree to which genetic and environmental factors contribute to myopia may vary depending on the age of onset.

Previous studies have reported that the primary risk factors associated with the onset and progression of myopia in adults are prolonged near work, shorter working distances, a higher AC/A ratio and increased accommodative lag [5–7]. The prevalence of myopia following near-work exposure has been reported to be significantly higher in adults than in children [8].

Prolonged near work has been consistently reported as a factor associated with myopia onset and progression, and evidence suggests that occupational exposure in adults may further contribute to its progression [8]. Since near work involves increased levels of accommodation and vergence, it is believed that these two systems play a role in the process that leads to changes in refractive error. To understand the role of these systems in myopia onset and progression, and how they may differ according to the age of onset, it is important to evaluate their function through the assessment of accommodation and vergence parameters. The majority of the studies have shown the relationship between accommodation and different types or levels of refractive error [9, 10], but there is a lack of clarity about accommodation and vergence in adults with early and late-onset myopia.

Early-onset myopia is normally developed in active ocular development and conditions of active near work like at school which can cause alterations in binocular functionality to adapt. Late-onset myopia, in contrast, occurs after ocular maturity, and it is usually related to occupational visual activities and other forms of accommodative vergence interactions. These developmental and environmental variations could be the cause of variations in parameters including amplitude of accommodation (AA), accommodative facility (AF), AC/A ratio, near point of convergence (NPC), vergence facility (VF), and fusional vergences. This comparison can be of great use in terms of understanding the binocular mechanisms that cause myopia and can be used in helping to implement individual clinical management approaches. This study aimed to assess and compare the accommodation and vergence parameters in early and late-onset myopic adults.

## Methods

This observational study was carried out at the Department of Ophthalmology, Kasturba Medical College and Optometry Clinic, Manipal. Participants were recruited from the Ophthalmology department through advertisements. The study proceeded after obtaining Institutional Research and Ethics Committee of Kasturba Hospital – Kasturba Medical College, Manipal Academy of Higher Education, Manipal (IEC number:281/2023), and adhered to the tenets of the Declaration of Helsinki. The Clinical Trial Registry-India was also obtained (CTRI/2023/08/056366; registered on 10/08/2023). The study included individuals aged 18–35 years with mild to moderate myopia. Informed consent was obtained from eligible participants before proceeding with the tests. Participants were divided into two groups: those with EOM (It is having the first spectacle or contact lens prescription before 15 years of age) and LOM (It is having the first refractive correction at 15 years of age or older). Participants underwent routine eye examinations, including visual acuity testing, subjective and objective refraction, and slit lamp examination. Various accommodation and vergence parameters, such as the amplitude of accommodation (AA), accommodative facility (AF), accommodative response, AC/A ratio, near point of convergence (NPC), positive fusional vergence (PFV), negative fusional vergence (NFV) and vergence facility (VF) were measured.

AA: Amplitude of accommodation is a measure of the closest point at which the eyes can focus. It is the range from the far point to the near point. AA was assessed both monocularly and binocularly using the push-up technique. The accommodative target (N8) was slowly pushed towards the participant’s eye until the first sustained blur was reported. The distance between the spectacle plane and the blur point was recorded in centimetres. This was performed three times. The average of these three values was recorded as the final value. The values measured in centimetres were then converted to diopters by dividing 100 by the obtained value.

AF: Accommodative facility is a subjective measure of an individual’s ability to rapidly and accurately alter their accommodative state. It was evaluated both monocularly and binocularly using a ± 2.00D accommodative flipper. A word rock card (N8) was held at 40 centimetres. An accommodative flipper was given to the participants. First, they were asked to keep the plus lens of the flipper in front of their eye and read words when they were clear and then flip to the minus lens. Focusing through a plus lens and a minus lens was considered as one cycle, and this was performed for one minute.

Accommodative response: It was measured objectively by dynamic retinoscopy using the MEM (Monocular Estimation Method). It is an objective method of measuring accommodative response at near when the patient is actively accommodating. It was performed using MEM card with printed letters held at 40 cm in a normal room illumination. Participants were instructed to read the words written on that card slowly. Based on the reflex, plus or minus lenses were used to neutralise the movement. After neutralising the reflex, lens power was recorded.

NPC: Near point of convergence is the nearest point at which an object is seen single when the lines of sight of the eyes intersect, with both eyes maximally converged. It was assessed using the accommodative target. The linear target was slowly pushed toward the participant until the participant first noticed diplopia. The distance between the canthus and the breakpoint was measured. This was performed three times, and the final NPC was recorded by taking the average of these three values.

NFV and PFV: Negative fusional vergence is the eyes’ ability to diverge to maintain single binocular vision, and negative fusional vergence is the ability to converge to maintain single binocular vision. It was evaluated for both distance and near. For near, participants were instructed to hold a linear target at a distance of 40 cm, and for the distance target was placed at 6 m. First, NFV was measured by keeping the prism base-in, in front of the participant’s one eye. The prism power was increased until consistent diplopia occurred. Then, PFV was measured by keeping the base-out prism in front of the participants’ one eye, and the prism power was increased until consistent diplopia occurred. The amount of prism when participants reported break and recovery was recorded.

VF: Vergence facility is measured in cycles per minute and reflects the ability of the fusional vergence system to respond rapidly and accurately to changing vergence demands. A vergence flipper (3∆ Base-in / 12∆ Base-out) was used to measure VF. A linear target was held at 40 cm. Participants were instructed to maintain a clear and single vision of the target. A vergence flipper was held in front of the participant’s eye, and once the letters became single and clear, it was flipped. When the letters were single and clear through 3∆ base-in and 12∆ base-out, it was recorded as one cycle. This was performed for one minute. The procedure was done binocularly.

AC/A ratio: It is the ratio of accommodative convergence (AC (in prism diopters)) to the stimulus to accommodation (A (in diopters)). It was calculated using the gradient method. The Modified Thorington test was used to measure near phoria at 40 cm. Then, again, near phoria was measured by keeping a -1.00 D lens in both eyes. The difference in the phoria with the added minus lens is recorded as the AC/A ratio.

### Sample size

The required sample size was calculated using the formula for comparison of two means. The standard deviation of the Amplitude of accommodation (1.065) was derived from a previous study [11]. A clinically significant difference of 0.9 was considered, with a significance level of 0.05 and power of 90%. Based on these parameters, the calculated sample size was 30 in each group (total = 60).

### Statistical analysis

Data was analysed using Jamovi 2.3.28 software. The normality of the data was checked using the Shapiro-Wilk test. Median ± IQR was reported as the data obtained from the study participants were not normally distributed. To compare accommodation and vergence parameters in early and late-onset myopia, the Mann-Whitney U test was performed. Chi-square test was used for categorical variables. P-value < 0.05 was considered statistically significant.

## Results

A total of 60 participants (30 early-onset myopic individuals and 30 late-onset myopic individuals) were included in the study. The mean age at presentation of the early-onset myopic individual was 21.8 ± 2.3 years, and the late-onset myopic individual was 21.9 ± 2.6 years. There was no statistically significant difference in the mean age at presentation between the two groups (*p* = 0.899). In the early-onset myopic group, 16.6% of the participants were males and 83.3% were females. Similarly, in the late-onset myopic group, 20% were males and 80% were females. The difference in gender distribution between the two groups was not statistically significant (*p* = 0.73). The mean age of the participants at myopia onset was 10.4 ± 2.6 years in the early-onset myopic group and 17.8 ± 2.4 years in the late-onset myopic group (Table 1). The study included individuals with mild to moderate myopia, while those with high myopia (>-6.0D) were excluded. In early-onset myopia group, the mean spherical equivalent (SE) was − 2.9 ± 1.3 D in right eye and − 2.7 ± 2.3 D in the left eye. In late-onset myopia group, the mean SE was − 1.7 ± 0.9 D in the right eye and − 1.4 ± 1.1D in the left.

**Table 1:** Demographic details of the participants

	Early-onset myopes	Late-onset myopes	*p*-value
Age (years)	21.8 ± 2.3	21.9 ± 2.6	0.899
Age of myopia onset (years)	10.4 ± 2.6	17.8 ± 2.4	< 0.001
Gender (male)	5 (16.6%)	6 (20%)	0.739
Gender (female)	25 (83.3%)	24 (80%)	

This study evaluated and compared the accommodation and vergence parameters between myopia with different ages of onset. Comparison between the groups was made using the Mann-Whitney U test.

For accommodative measures, the amplitude of accommodation was lower in the late-onset myopes compared with early-onset myopes across right eye, left eye and binocular measures. However, these differences were not statistically significant (*p* > 0.05). In contrast, the accommodative facility (AF) demonstrated a significant difference between the EOM and LOM groups indicating slower accommodative response and reduced flexibility of the accommodative system in adults who developed myopia later. AF was reduced in late-onset myopes compared with early-onset myopes both monocularly and binocularly (OD: 8.50 ± 4.75 vs. 12.5 ± 4.50 cpm, *p* < 0.001; OS: 8.50 ± 4.13 vs. 12.0 ± 5.00 cpm, *p* = 0.001; OU: 9.25 ± 4.88 vs. 12.8 ± 4.38 cpm, *p* = 0.002;). Lower accommodative facility indicates reduced ability to rapidly and accurately alter their accommodative state, suggesting that LOM have less adaptable accommodative function. In MEM retinoscopy, no statistically significant differences were found between early and late-onset myopes (*p* > 0.05). However, both groups demonstrated a lag of accommodation (Table 2).

**Table 2:** Comparison of accommodative parameters between EOM and LOM

Parameters	Type of onset	Median ± IQR	*p*-value
AA_OD (D)	Early-onset	13.4 ± 6.6	0.089
	Late-onset	11.1 ± 4.0	
AA_OS (D)	Early-onset	12.5 ± 6.6	0.06
	Late-onset	10.0 ± 3.7	
AA_OU (D)	Early-onset	14.3 ± 5.5	0.053
	Late-onset	12.5 ± 3.5	
AF_OD (cpm)	Early-onset	12.5 ± 4.5	**< 0.001**
	Late-onset	8.5 ± 4.7	
AF_OS (cpm)	Early-onset	12.0 ± 5.0	**0.001**
	Late-onset	8.5 ± 4.1	
AF_OU (cpm)	Early-onset	12.8 ± 4.3	**0.002**
	Late-onset	9.2 ± 4.8	
MEM_OD (D)	Early-onset	0.7 ± 0.2	0.061
	Late-onset	1.0 ± 0.4	
MEM_OS (D)	Early-onset	1.0 ± 0.2	0.171
	Late-onset	1.0 ± 0.5	

AA: Amplitude of accommodation, AF: Accommodative facility, MEM: Monocular Estimation Method

OD: Right eye, OS: Left eye, OU: Both eyes

The AC/A ratio was higher in the late-onset myopia group (3.5 ± 2.0) compared with the early-onset myopia group (2.0 ± 1.0), although the difference was not statistically significant (*p* > 0.05).

In vergence measures, there was no significant difference in the near point of convergence (*p* > 0.05). Similarly, the vergence facility showed no significant difference between early and late-onset groups. However, both negative fusional vergence and positive fusional vergence for distance showed statistically significant differences between the early and late-onset myopes. Late-onset myopes demonstrated lower negative fusional vergence at distance (break: 7.0 ± 5.5 vs. 10.0 ± 4.0 PD, *p* = 0.002; recovery: 6.0 ± 5.5 vs. 8.0 ± 4.0 PD, *p* = 0.005;) and positive fusional vergence at distance (break: 9.0 ± 8.0 vs. 12.0 ± 11.0 PD, *p* = 0.018; recovery: 7.0 ± 8.0 vs. 10.0 ± 11.0 PD, *p* = 0.024) suggesting weaker convergence reserves and potentially less stable binocular vision during distance viewing in late-onset myopia. Lower positive or negative fusional vergence indicate a reduced ability to diverge or converge to maintain single binocular vision at distance, suggesting that LOM have less stable binocular vision than EOM (Table 3).

**Table 3:** Comparison of vergence parameters between EOM and LOM

Parameters	Type of onset	Median ± IQR	*p*-value
NPC_BREAK (cm)	Early-onset	7.0 ± 2.5	0.140
	Late-onset	7.5 ± 4.0	
NPC_RECOVERY (cm)	Early-onset	9.0 ± 3.0	0.367
	Late-onset	11.0 ± 5.0	
VF (cpm)	Early-onset	13.3 ± 3.7	0.102
	Late-onset	12.0 ± 3.0	
NFV_D_BREAK (PD)	Early-onset	10.0 ± 4.0	**0.002**
	Late-onset	7.0 ± 5.5	
NFV_D_RECOVERY (PD)	Early-onset	8.0 ± 4.0	**0.005**
	Late-onset	6.0 ± 5.5	
NFV_N_BREAK (PD)	Early-onset	12.0 ± 2.0	0.133
	Late-onset	11.0 ± 4.0	
NFV_N_RECOVERY (PD)	Early-onset	10.0 ± 2.0	0.150
	Late-onset	9.0 ± 4.0	
PFV_D_BREAK (PD)	Early-onset	12.0 ± 11.0	**0.018**
	Late-onset	9.0 ± 8.0	
PFV_D_RECOVERY (PD)	Early-onset	10.0 ± 11.0	**0.024**
	Late-onset	7.0 ± 8.0	
PFV_N_BREAK (PD)	Early-onset	15.0 ± 10.0	0.071
	Late-onset	12.0 ± 1.5	
PFV_N_RECOVERY (PD)	Early-onset	13.0 ± 10.0	0.060
	Late-onset	10.0 ± 2.0	
AC/A_RATIO	Early-onset	2.0 ± 1.0	0.072
	Late-onset	3.5 ± 2.0	

NPC: Near point of convergence, VF: Vergence facility, NFV: Negative fusional vergence, PFV: Positive fusional vergence

D: Distance, N: Near

Overall, significant differences were observed in AF, NFV and PFV for the distance between early and late-onset myopic individuals, with late-onset myopic individuals showing reduced AF, NFV and PFV values compared to early-onset myopic individuals. These differences suggest that adults with late-onset myopia may experience reduced accommodative adaptability and vergence flexibility, which could contribute to visual fatigue during prolonged near tasks.

A Spearman’s correlation analysis was performed to explore the association between the onset category EOM and LOM, accommodative parameters, and vergence functions. A significant negative correlation was observed between the myopia onset category and accommodative facility (*r* = − 0.414, *p* = 0.001), indicating that LOM exhibited reduced accommodative flexibility compared to EOM. Similarly, the onset category demonstrated a significant negative correlation with distance negative fusional vergence (NFV) break (*r* = − 0.403, *p* = 0.001) and distance NFV recovery (*r* = − 0.370, *p* = 0.004), suggesting poorer divergence control among LOM. No significant associations were found between the onset category and accommodation amplitude (*r* = − 0.253, *p* = 0.051) or positive fusional vergence (PFV) parameters (*p* > 0.05). Although weak positive correlations were observed between accommodation amplitude and both PFV and NFV measures, these did not reach statistical significance. Strong positive correlations were noted between vergence break and recovery values within each vergence type (NFV and PFV at near and distance; all *p* < 0.001), indicating good internal consistency among vergence measures.

## Discussion

This study compared the accommodation and vergence parameters between myopes with different ages of onset. The findings revealed that late-onset myopic individuals demonstrated reduced monocular and binocular accommodative facility compared to early-onset myopes, suggesting that the age at onset of myopia may influence accommodative flexibility. Pandian et al. (2006), who compared accommodative facility among myopes, emmetropes, and hyperopes, reported no significant difference in near facility but observed reduced distance facility in myopes [12]. Although the groupings are not directly comparable, both studies highlight that accommodative facility can vary with refractive status or onset characteristics.

When accommodative lag was assessed, no significant difference was observed between early- and late-onset groups in the present study. However, both groups exhibited a lag of accommodation, indicating that accommodative lag is associated with myopia itself rather than the age of onset which is consistent with earlier evidence indicating increased accommodative lag in myopic individuals [13]. 

No significant difference was found in the amplitude of accommodation between the two onset groups. In contrast, McBrien (1986) reported higher accommodative amplitudes in late-onset myopes, followed by early-onset myopes, emmetropes, and hyperopes [11].The difference could be due to the variations in characteristics of the population involved or due to the study design. This suggests that further study is required to understand how the age of myopia onset influences accommodation.

The present study also found no significant difference in the AC/A ratio between the two groups. Rosenfield and Gilmartin (1987) explored the impact of near-vision tasks on AC/A ratio in emmetropes, early-onset myopes and late-onset myopes. They found that early-onset myopes had a significantly higher response AC/A ratio compared to emmetropes and late-onset myopes [14]. This variation may be due to differences in study design or stimulus conditions.

The present study also revealed that late-onset myopic individuals have reduced negative and positive fusional vergence at distance compared to early-onset myopic individuals. However, no difference was found between the two groups in negative and positive fusional vergence for near.

The reduced accommodative facility and distance fusional vergence in late-onset myopes may be due to differences in visual system adaptability. Myopia developing early in life occurs during a period of high neural plasticity, enabling gradual adaptation to blur and sustained near work, leading to stronger accommodative and vergence control. Late-onset myopes, on the other hand, face the difficulties of myopia after visual maturity, when neural plasticity is lower and the visual system has less capacity to adjust. Furthermore, late-onset myopes are exposed to shorter periods of myopic blur, experience higher near vision demands during adulthood, which may lead to reduced accommodative responsiveness and distance vergence ranges [15]. 

Another factor which might have caused the decreasing accommodative facility and distance fusional vergence in late-onset myopes is the high visual demand usually experienced during early adulthood. This phase is commonly associated with long near vision activities like reading, studying or frequent use of the screen, which may put a considerable stress on the accommodative and vergence systems [16]. Such high near vision requirements can exceed the adaptive capacity of accommodation and fusional vergence, resulting in less flexibility and performance in individuals whose visual system has already matured. Conversely, early-onset myopes, whose visual systems have developed with persistent exposure to myopic blur [15], can eventually adjust to these requirements over time, and thus have stronger accommodative and vergence responses. Thus, the poorer accommodative facility and distance vergence function of late-onset myopic individual may be partly due to lifestyle and visual demand factors.

Correlation analysis in the present study further supports these differences. Significant negative correlations were observed between the onset of myopia and both accommodative facility and distance NFV parameters, indicating reduced accommodative and divergence flexibility in late-onset myopes. This aligns with previous findings showing that early-onset myopes exhibit more efficient oculomotor adaptation due to long-term near-work exposure and earlier development of accommodative–vergence linkages [17, 18]. The reduced NFV reserves and slower recovery among late-onset myopes may reflect a less efficient fusional vergence mechanism, which could increase vergence demand and contribute to symptoms of visual fatigue during sustained near work [19, 20].

Although accommodation amplitude correlated weakly and non-significantly with vergence measures, the positive trend observed suggests an interdependence between these systems, consistent with the concept of accommodative–vergence adaptation [21, 22]. However, the lack of significance may indicate partial dissociation of the two systems in myopia, possibly due to neural recalibration with increasing refractive error [23]. The strong correlations observed between break and recovery values across vergence parameters confirm the internal consistency and reliability of these binocular measurements.

Overall, these findings suggest that early-onset myopes demonstrate superior accommodative facility and divergence ability compared to late-onset myopes, indicating more efficient binocular adaptive mechanisms. Clinically, these results emphasize the importance of assessing accommodative and vergence function in myopia management, as deficits in these systems may reduce visual comfort and influence the success of myopia control interventions.

The main limitation of this study is the possibility of recall bias, as participants may not accurately remember the exact age of myopia onset. Also, potential confounding variables such as duration of near work, education level, and gender distribution were not controlled, which could have influenced the outcomes. Future longitudinal or regression-based studies with larger sample sizes and controlled covariates are warranted to confirm these associations.

## Conclusion

This study identified significant differences in monocular and binocular accommodative facility and distance fusional vergence between early- and late-onset myopic adults. Late-onset myopes presented poorer accommodative facility and reduced distance fusional vergence compared to early-onset myopes, although both groups showed a lag of accommodation.

These findings indicate that the age of myopia onset may influence visual performance and binocular coordination. Therefore, clinicians should consider onset characteristics during examination and treatment planning. A detailed assessment of accommodative and vergence anomalies in late-onset myopes is essential to reduce visual fatigue and improve visual comfort. Individualized vision therapy or targeted binocular training may be beneficial in managing these deficits. Future studies involving larger populations and accounting for visual demand and lifestyle factors will help strengthen the evidence base for tailored interventions.

## Data Availability

The datasets used and/or analysed during the current study are available from the corresponding author on reasonable request.Point of contact for data availability is Salai Dhavamathi J, email id is dhavamathi.j@manipal.edu.

## Code availability

NA.
